# Dirty Money on Holy Ground: Isolation of Potentially Pathogenic Bacteria and Fungi on Money Collected from Church Offerings

**Published:** 2019-05

**Authors:** Akebe Luther King ABIA, Eunice UBOMBA-JASWA

**Affiliations:** 1. Antimicrobial Research Unit, College of Health Sciences, University of KwaZulu-Natal, Private Bag X54001, Durban 4000, South Africa; 2. Department of Biotechnology, University of Johannesburg, Gauteng, South Africa; 3. Water Research Commission, Pretoria, South Africa

**Keywords:** Money, Disease transmission, Church, Microbial contamination, Public health

## Abstract

**Background:**

Fomites (including money) can transmit diseases to humans. How the nature of money influences contamination has not been adequately demonstrated. Moreover, such studies in church settings are non-existent. Thus, we studied how money collected from a church could serve as human disease transmission vehicles.

**Methods:**

Overall, 284 money samples (currency notes and coins) were collected during two Sundays in the months of Nov and Dec 2015 from a church congregation in Pretoria, Gauteng, South Africa. The presence of potentially pathogenic bacteria and fungi were investigated using culture (Colilert^®^ method) and molecular methods (Sanger sequencing). Scanning Electron Microscopy (SEM) was used to visualize the possible positions of the bacteria on various parts of a currency note.

**Results:**

Of the 192 samples (first sampling round), 76 (39.6%) were positive for *E. coli*. Smaller notes (R10) recorded the highest *E. coli* counts per note. Of the 92 notes analyzed for potentially pathogenic bacteria and fungi (second sampling round), 76 (82%) showed growth on at least one of the six culture media used. Sequencing revealed three bacterial (*Bacillus*, *Staphylococcus* and *Corynebacterium*) and two fungal (*Clavispora* and *Rhodotorula*) genera. SEM revealed that microorganisms could enter cracks of creased notes.

**Conclusion:**

Unlike previous studies conducted where recent contamination could occur, the current study shows that microorganisms can survive on money; samples were collected from a church, where little or no exchange takes place. Moreover, using SEM demonstrates that aged and creased notes favor attachment of bacteria to money and could be of public health concern by transmitting disease within a given population.

## Introduction

Although pathogens could only survive in their hosts, some pathogens can survive and multiply outside of their natural hosts (1). Living hosts provide microorganisms with adequate food, moisture and temperature favorable for their survival (2,3). Similar conditions are seldom found on non-living things. Nevertheless, pathogens have been isolated from inanimate objects which can serve as vehicles for their transmission between hosts (1). One of such objects includes money (4–6). Some factors promoting the contamination of money include the age of the money (duration in circulation), users’ socio-economic status, the currency denomination and the material used to make the money (5,7). Cotton-based currency notes contained three times higher bacterial counts than polymer-based ones (7), concluding that the spaces between the cotton fibers provide favorable anchor (unlike with polymer materials) for the bacteria. Polymers are preferred over cotton and paper materials because polymers allow for durability and easy incorporation of security features to avoid counterfeiting (8). The South African paper currency, for example, is made of a unique paper allowing for raised printing as a security feature (9). Such features could provide settling spaces for microorganisms and debris, thus favoring the survival of the settled organisms (5).

Most developed countries have almost entirely shifted from hard “physical cash” to virtual money as in credit cards. Many developing countries, however, still depend on physical cash for exchange, for both small and high-value items (7). As the money moves from person-to-person, it gets contaminated with diverse microorganisms. Studies in recent years have isolated different organisms on currencies around the world (4). The microbial quality of money collected from food outlets in 10 different countries, both developed and developing, were examined and it was found that some bank notes in the USA, for example, contained up to 2.5 ×10^4^ CFU/note (total bacterial count) (7). Similarly, 93.9% of Cameroonian bank notes and coins analyzed in a study contained diverse microorganisms including pathogens (10). Most of these studies, however, were conducted in areas where it is evident that contamination would occur, like with food vendors, hospital cashiers, and taxi drivers, given the frequent exchange of money in these places. One place given little or no attention for such studies and scientific research, in general, is “the holy ground” or the “church”.

Money also forms an integral part of churches globally. During church gatherings, some important elements are offerings and tithing (11). Interestingly, a worshipping congregation consists of individuals from all walks of life. Therefore, the church could be regarded as a microcosm of society, and, the microbial evaluation of money collected in the church could give insight into the microbial diversity within a community.

Thus, we used culture, molecular methods and SEM to investigate the microbial quality of money collected from offering baskets in a congregation in Pretoria, South Africa.

## Materials and Methods

### Sample collection

Overall, 284 money samples (currency notes and coins) were collected during two Sundays in Nov and Dec 2015 from a church congregation in Pretoria, Gauteng, South Africa. Samples were collected from the offering baskets after the counting by the church’s financial department (who were also church members), put into sterile zip-lock bags by those who counted (to avoid possible contamination from the researchers) and transported to the laboratory for microbial analysis. On each of the sampling days, information on the various professions of the church attendants was collected.

Before sampling, verbal informed consent was obtained from the church’s administration. Based on the information sheet obtained from the church’s attendants, the members of the church included consultants, house helpers, students, gender experts, security guards, researchers, unemployed, hairdressers, dressmakers, panel beaters, mechanics, plumbers, miners, electricians, lecturers, auditors, and clergies.

### Microbial analysis

#### Enumeration of E. coli on currency notes and coins

Overall, 192 samples (coins and paper notes) were collected during the first sampling round (Nov 2015) and analyzed for *E. coli* using the Colilert^®^ method (12). Each sample was transferred into a 120-mL cup containing 50 mL 0.1× sterile phosphate buffered saline (PBS), clamped to a Heidolph REAX 20/8 mixer shaker (Heidolph Instruments, GmbH & Co., Germany) and set to rotate at 15 rpm/min for 45 min to wash off the microorganisms from the samples. Each cup was then topped up to 100 mL with sterile PBS and analyzed using the Colilert^®^ 18/Quanti-Tray^®^ system following the manufacturer’s instructions (13). Results were recorded as most probable number (MPN) per note or coin.

#### Isolation of potentially pathogenic bacteria and fungi from currency notes

Isolation of potentially pathogenic bacteria and fungi was done on 92 samples (notes only; all coins were negative for *E. coli* in the first round and so were excluded) collected during the second sampling round (Dec 2015). The currency notes were in different physical states: newly minted, dirty and some creased and torn notes. The surface of each note was swabbed (10) and streaked onto five different culture media. For bacteria, swabs were streaked on Nutrient Agar (NA; Merck, South Africa), Cereus Selective Agar (CSA; Fluka Analytical, South Africa), Pseudomonas Isolation Agar (PIA; Sigma-Aldrich, South Africa), Membrane Lactose Glucuronide Agar (MLGA; Oxoid, South Africa) and Salmonella-Shigella Agar (SSA; Merck, South Africa). For fungi, the swabs were streaked on Rose Bengal Agar with chloramphenicol (RBA; CONDA, South Africa). The plates for bacteria isolation were incubated at 35 °C for 24 h. The RBA plates were incubated at 22 ± 2 °C for five days.

#### Sequencing of isolates for identification of bacterial and fungal species

Based on morphological characteristics, the most frequently isolated organisms on culture plate were selected (eight bacterial and two fungal colonies) and sent to Inqaba Biotechnology, South Africa, for sequencing using the Sanger method. DNA was extracted from the cultures using the ZR Fungal/Bacterial DNA Kit^TM^ (Zymo Research Corp., Irvine, CA, USA). The 16S target region (for bacteria) was amplified using DreamTaq^TM^ DNA polymerase (Thermo Scientific^TM^, Johannesburg, South Africa) and universal primers 27-Forward and 1492-Reverse (14). Two isolates, morphologically identified as either fungi or yeast on RBA, were sequenced targeting the internal transcribed spacer (ITS) using primers ITS1 and ITS4 (15). PCR amplicons were gel-extracted using the Zymoclean^TM^ Gel Recovery Kit (Zymo Research Corp.) and sequenced in the forward and reverse directions on an ABI PRISMTM 3500×l Genetic Analyser. The sequencing products were purified using the ZR-96 DNA Sequencing Clean-up kit^TM^ (Zymo Research Corp.) and analyzed using the CLC Main Workbench v7.6 followed by a BLAST (NCBI) (16).

#### Scanning electron microscopy (SEM) of currency notes

To check if creased notes possessed cracks wide enough to harbor bacteria, an old R10 note was subjected to SEM. An intact portion and a creased portion of the note were observed. Later, a fresh overnight culture of *E. coli* (ATCC 25922; American Type Culture Collection, Manassas, VA, USA) was inoculated by spreading 100 μL of broth culture using a sterile glass spreader, on the surface of the R10 note. The inoculated note was sent to the Electron Microscopy Unit of the Council for Scientific and Industrial Research (CSIR), South Africa for observation using SEM. This was to view whether these microorganisms remained on the surface of the money or were trapped in the cracks of creased notes.

### Statistical analysis

The mean *E. coli* concentrations of the various currency denominations were compared for statistical differences using ANOVA (one-way analysis of variance). Levene’s statistic was used to test for homogeneity of variance of the various denominations. Based on the Levene’s test, the different denominations were of different sample sizes, the Games-Howell post-hoc test was performed to check for the groups accounting for the overall significant difference observed. All tests were performed using SPSS, ver. 20.0 (IBM Corporation, New York, USA). Results were considered statistically significant α = 0.05.

## Results

### Microbial analysis

Overall, of the 192 money samples collected (currency notes and coins), 76 (39.6%) were positive for *E. coli* (Table 1).

**Table 1:** Mean *E. coli* concentration (MPN) per denomination (first sampling round)

	***Denomination***	***No. of samples analyzed***	***No. of samples positive***	***Minimum E. coli count/currency note***	***Maximum E. coli count/currency note***	***Mean E. coli count/currency note***
Currency notes	R10	74	53	1	42.5	11.9
	R20	46	12	1	18.9	5.3
	R50	30	6	2	7.5	3.9
	R100	15	5	1	5.2	2.7
	R200	4	0	/	/	/
Coins	50c	8	0	/	/	/
	20c	11	0	/	/	/
	10c	4	0	/	/	/

The single highest *E. coli* concentration (42.5 MPN) per sample was obtained on a R10. All coins and R200 notes were negative for *E. coli*. Regarding the proportion of positive samples to the number of samples collected per denomination, the R10 notes were more contaminated (71.6%) than the R20 (26.1%), R50 (20%), R100 (33.3%) and the R200 (0%). An ANOVA test revealed a statistically significant difference (*P*=0.001; *P*<0.05) between the mean *E. coli* concentrations on the various denominations. Specifically, the mean *E. coli* concentration on the R10 was statistically significantly higher compared to the R20 (*P*=0.017; *P*<0.05), R50 (*P*=0.000; *P*<0.05) and R100 (*P*=0.000; *P*<0.05). No statistically significant difference was observed between the mean *E. coli* concentrations on the R20 and R50 (*P*=0.271; *P*>0.05); R20 and R100 (*P*=0.095; *P*>0.05), and between R50 and R100 (*P*=0.684; *P*<0.05).

Of the 92 currency notes examined for potentially pathogenic bacteria and fungi, 76 (82%) showed growth on at least one of the six culture media used (Table 2). The highest growth was observed on NA. No growth was recorded on MLGA.

**Table 2:** Number of samples showing growth on the various culture media used

***Number of positive samples***							

***Denomination***	***No. of Samples***	***NA***	***MLGA***	***CSA***	***PIA***	***RBA***	***SSA***
R10	49	41	0	35	14	20	15
R20	24	20	0	18	9	7	3
R50	12	10	0	7	2	2	0
R100	7	5	0	2	0	1	0

NA=Nutrient Agar; MLGA=Membrane Lactose Glucuronide Agar; CSA=Cereus Selective Agar; PIA=Pseudomonas Isolation Agar; RBA=Rose Bengal Agar; SSA=Salmonella-Shigella Agar.

N.B.: Numbers in each agar type represent the number of positive samples and not the number of colonies isolated.

The most isolated microorganisms, based on sequencing, were from the genera *Bacillus*, *Staphylococcus* and *Corynebacterium*, (for the bacteria) and *Clavispora* and *Rhodotorula* (for the fungi) (Table 3). Of these, the most isolated bacteria were in the genus *Bacillus*.

**Table 3:** BLAST prediction for the various isolates sequenced

***Denomination***	***Blast prediction***
R10	*Bacillus* sp.
R10	*Bacillus subtilis*
R10	*Staphylococcus cohnii*
R10	*Bacillus* sp.
R10	*Bacillus subtilis*
R10	*Bacillus thuringiensis*
R20	*Corynebacterium* sp.
R50	*Bacillus subtilis*
R10 – ITS Target	*Clavispora lusitaniae*
R100 – ITS Target	*Rhodotorula mucilaginosa*

### Scanning electron microscopy of currency notes

**Fig. 1:**
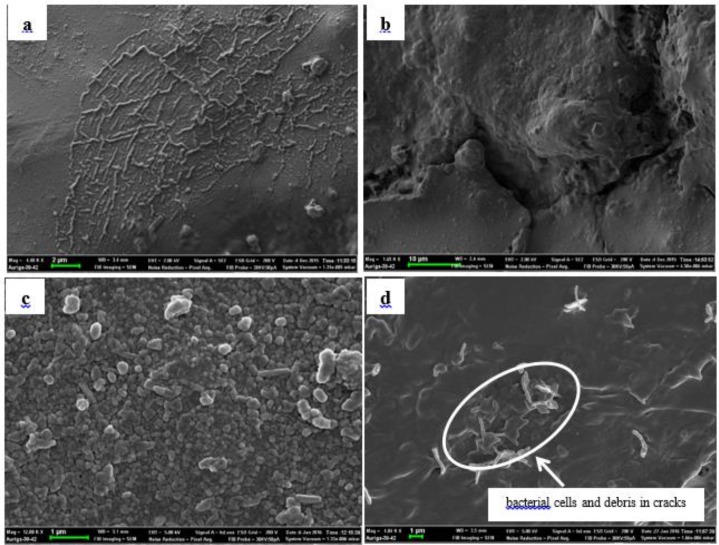
Electron micrographs of a South African R10 currency note: (a) uninoculated intact portion, (b) uninoculated creased portion (c) inoculated intact surface and (d) inoculated creased surface

Figure 1 illustrates the surface of an intact portion of a R10 note (a) and that of a creased portion (b). The microorganism could either stay on the surface of intact notes (c) or enter the cracks on creased notes (d).

## Discussion

### Microbial analysis

Fomites have been recognized for their disease transmission potentials to humans (17). In the current study, 154/284 (53.5%) samples collected during both sampling rounds were positive for bacteria and/or fungi. The detection of microorganisms on the samples in this study reiterates the fact that money could serve as a vehicle for disease transmission as earlier suggested by studies conducted in other countries (5,7,10,18). The overall percentage of positive samples in the current study were lower than those recorded in some earlier studies. For example, 98.4%, 87.3% and 79.4% for heterotrophic plate count, coliform bacteria and streptococci were recorded, respectively (19). Similarly, an overall 93.9% contamination was previously reported, with bank notes recording higher contamination percentages (96.6%) than coins (88.2%)(10).

Also, the overall percentage of currency notes positive for *E. coli* (39.6%) was slightly lower than the 41.7% obtained on Indian notes and coins (20). Our results also contradict those of Singh and colleagues in that, unlike the 35.41% contaminated coins obtained in their study, all coins analyzed in our study were negative for *E. coli*. This could have been influenced by the type of materials used for making the various coins. Indian coins are made of ferritic stainless steel (21) which possess minimal antibacterial effects (22). Contrary, the South African coins are made of Nickel-plated copper, bronze-plated steel or copper-plated steel (9). Nickel, copper, and bronze (a copper alloy) all possess antibacterial properties (22–23). Moreover, unlike other studies where the coins were mostly used by communities, in South Africa, the cents are not common in circulation as they have a low value in relation to goods to be purchased. Thus, many people do not use the cents daily, contributing to the non-detection of bacterial contamination on these samples in the present study. Contrary to the other studies on samples from points like food vendors (7), where recent contamination could occur due to the frequent exchange of money at such points, samples in the current study were collected from a church basket on a day when no buying nor selling activities took place and minimal exchange occurred. This difference in sampling points could have also influenced the lower microbial contamination observed in the current study compared to these previous studies. Although those counting the money could also contaminate the money, such contamination would not be expected to be higher than in settings like food outlets. The finding that the lower denominations of the currency notes were more contaminated than the higher ones in this study (*P*<0.05) corroborate other findings (10). The rate of contamination increased from higher to lower notes, suggesting that lower notes were more in circulation than the larger ones, thus had a greater chance of contamination.

The lack of bacterial growth on MLGA during the second sampling round (compared to the 76 positive samples obtained in the first sampling round) was unexpected since the media is selective for *E. coli* and coliforms. This could have been due to the different methods used. In the first round, samples were thoroughly rinsed, thus maximizing the chances of washing off the bacteria from the money samples. Moreover, the whole rinsate was analyzed for *E. coli* alone. Contrarily, in the second round, a single swab was used to inoculate the various media. This might have decreased the bacteria concentration when inoculating from one media type to the next.

Identification of *Bacillus* spp. as the most isolated organisms on currency notes corroborates other findings (10,20). *Bacillus* spp. form spores enhancing their survival in harsh environmental conditions (24). This spore-forming ability could have therefore led to *Bacillus* spp. being the most isolated bacteria in the study. Some strains of the organisms isolated and sequenced in the second sampling round are known to be pathogenic. Some *Bacillus* spp. are the etiology agents of human diseases like anthrax (24,25) and in some cases have resulted in fatal sepsis in immunocompromised individuals (26–28). *Staphylococcus cohnii*, causes bacteremia in patients with catheters and surgical prostheses (28). The organism has also been implicated in intrascrotal abscesses (29), sepsis, and nosocomial meningitis in new-borns (30). Members of the genus *Corynebacterium* cause urethritis, respiratory and skin infections (31–33). These organisms (corynebacteria) are increasingly being recognized as emerging opportunistic pathogens (34–35).

The fungi isolated in the current study were also pathogenic as reported in literature. *Clavispora lusitaniae*, has been isolated in fungemias and meningitis cases in hematologic malignancies patients (36). *Rhodotorula mucilaginosa* is an emerging opportunistic pathogen implicated in catheter-related fungemia in leukemic children (37), lymphadenitis in HIV patients (38), keratitis (39) and persistent femoral non-union (40). Most of these pathogens are related to urinary infections, suggesting that the most likely form of contamination of the money was poor hygienic practices after using the toilets. This could pose a potential health threat to immunocompromised individuals and children. Commonly observed in the congregation during sample collection was the giving of coins to children to play with, to prevent them from disturbing the service. The children often put the money into their mouths. Moreover, the changing of children’s diapers and feeding children in church are some of the practices that could lead to infection of the children. Microorganisms on fomites could be transferred to fingers at an approximate 7%–80% transfer efficiency (1). Some women were also observed keeping items like mobile phones and money in their bras, another practice that could transfer microorganisms between the skin and money. The transfer of skin pathogens onto items like fabrics and vice versa has been reported (41). Such practices could provide moisture and warmth from sweating bodies, creating conditions favorable for the survival of microorganisms on the money. Thus, these microorganisms could be the transfer to children during breastfeeding, if proper hygiene is not observed. Such practices could also lead to skin infections like those involving some members of the genus *Staphylococcus* (42).

### Scanning electron microscopy of currency notes

Some factors favoring contamination of money include the physical state of the note and the material used for making the notes (7,10). Creased portions form openings on the surface of the currency note (Fig. 1). Thus, in the intact nature, microorganisms will remain on the surface of the South African Rand. The polymer nature of the South African paper money, makes it possible for cracks to occur on the money when creased, thus creating favorable hiding spaces for microorganisms. The cracks formed a similar environment like those observed on cotton-based currency notes (e.g. the British Pound) as previously described (7).

## Conclusion

The microorganisms could have survived on the money; samples were collected from a church, where little exchange takes place. SEM demonstrated that aged and creased notes favor bacterial attachment to money and could be of public health concern by transmitting disease within a population. Women keeping money on body parts, children and immunocompromised individuals could be at higher risk of infection. Therefore, it is important to promote hygienic practices within the population. Improving the antibacterial properties of materials used to make money could prevent transmission of potential pathogens.

## Ethical considerations

Ethical issues (Including plagiarism, informed consent, misconduct, data fabrication and/or falsification, double publication and/or submission, redundancy, etc.) have been completely observed by the authors.
